# Ovarian Torsion in the Third Trimester of Pregnancy Leading to Iatrogenic Preterm Delivery

**DOI:** 10.1155/2016/8426270

**Published:** 2016-03-15

**Authors:** Evangelia Vlachodimitropoulou Koumoutsea, Manish Gupta, Antony Hollingworth, Anwen Gorry

**Affiliations:** Department of Obstetrics and Gynaecology, Whipps Cross University Hospital, London E11 1NR, UK

## Abstract

Ovarian torsion in the third trimester of pregnancy leading to a midline laparotomy and caesarean section for the delivery of a preterm baby is an uncommon event. As the woman is likely to present with nonspecific symptoms of lower abdominal pain, nausea, and vomiting, ovarian torsion can often be misdiagnosed as appendicitis or preterm labour. Treatment and the opportunity to preserve the tube and ovary may consequently be delayed. We report the case of a multiparous woman who had undergone two previous caesarean sections at term, presenting at 35 weeks of gestation with a presumptive diagnosis of acute appendicitis. Ultrasonography described a cystic lesion 6 × 3 cm in the right adnexa, potentially a degenerating fibroid or a torted right ovary. MRI of the pelvis was unable to provide further clarity. The patient was managed by midline laparotomy and simultaneous detorsion of the ovarian pedicle and ovarian cystectomy together with caesarean section of a preterm infant. This report describes that prompt recognition and ensuring intraoperative access can achieve a successful maternal and fetal outcome in this rare and difficult scenario. Furthermore, we would like to emphasise that the risk for a pregnant woman and her newborn could be reduced by earlier diagnosis and management of ovarian masses (Krishnan et al., 2011).

## 1. Case Presentation

A 33-year-old woman was booked for hospital care because of two previous caesarean deliveries. The first was an emergency caesarean at 42 weeks of gestation for fetal distress in labour. The second was undertaken for failure to progress in spontaneous labour. In this pregnancy her last ultrasound scan was at 20 weeks of gestation and revealed no fetal abnormalities.

The patient presented at 35 + 2 weeks of gestation, with a 4-hour history of sudden onset and severe and constant abdominal pain in the right iliac fossa. She found changing position incredibly painful and examination displayed involuntary guarding and rigidity of the right side of her abdomen. The pain was associated with uncontrollable vomiting. There was no history of vaginal loss or bleeding and normal fetal movements had been felt.

## 2. Investigations

On examination, the patient was in obvious distress. She was normotensive and tachycardic; pulse rate was 110 bpm; respiratory rate was 16/min; and oxygen saturations were 100% in air. She was afebrile. Abdominal palpation revealed an exquisitely tender abdomen with rigidity and guarding on the lower right side. Acute appendicitis was suspected and a prompt review by the surgical team was undertaken.

Ultrasound assessment on the labour ward demonstrated fetal heart movements, cephalic presentation, and an anterior high lying placenta. Cervical length was 32 mm. Fetal monitoring using cardiotocography was reassuring.

The patient was managed conservatively overnight, was nil by mouth, and required high doses of oral morphine and antiemetics. A pelvic ultrasound scan revealed a right sided 6 × 3 cm cystic lesion, consistent with a degenerating fibroid or a torted ovary. A previous USS of the abdomen 3 years earlier commented on a 3 cm right ovarian dermoid cyst. The patient subsequently had a prompt MRI scan, though it was unable to provide further clarification on the aetiology of the pain. With the clinical presentation of an acute abdomen and severe vomiting requiring regular need for analgesia, the decision for a midline laparotomy was made. Given the patient's obstetric history of two previous caesarean sections, the decision for an emergency caesarean section was made by the obstetric and the paediatric consultant. The patient had had one dose of dexamethasone injection a few hours prior to delivery.

## 3. Differential Diagnosis

Differential diagnoses included appendicitis, degenerating fibroid, and ovarian torsion.

## 4. Treatment

**Figure 1:**
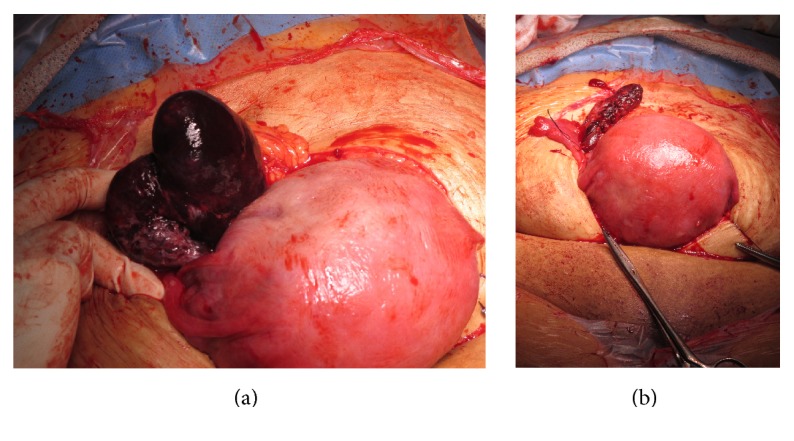
(a) Right ovarian cyst torsion. (b) Right ovary following resection of torted ovarian cyst.

Consent was taken for a category 2 emergency caesarean section. On opening the abdominal cavity through a midline laparotomy incision, a large purple but not necrotic right sided mass was noted. The caesarean section was performed initially, delivering a female infant. Apgars were 9 and 10 at 5 and 10 minutes and she was transferred to the neonatal unit for assistance with breathing and observation. The placental delivery was by controlled cord traction. Syntocinon infusion was commenced due to uterine atony. The uterus was closed in two layers and a number of haemostatic sutures were required at the midline. The right ovary was then examined and it was torted twice and appeared as a purple enlarged structure of 7 × 4 cm (Figure 1(a)). There were some well perfused white parts noted on the ovary on close examination. A cystectomy of the right dermoid and evacuation of blood clots were performed and the right tube and ovary were conserved (Figure 1(b)). Interestingly there was a 2 cm ovarian cyst, dermoid in appearance on the left ovary. Decision was made against resection. Closure of the abdomen was then completed routinely. The total intraoperative blood loss was 600 mL.

## 5. Outcome and Follow-Up

**Figure 2:**
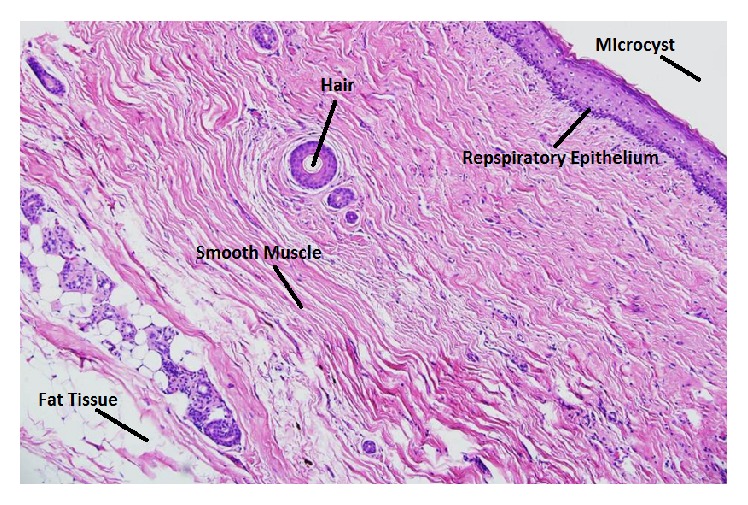
Ovarian dermoid histology (hematoxylin and eosin stain).

The patient made a good recovery and histological examination of the ovarian cyst confirmed a dermoid cyst (Figure 2). Further follow-up was arranged in the gynaecology clinic for surveillance of the contralateral cyst. The baby suffered no short or medium term complications from the effects of prematurity.

## 6. Discussion and Conclusion

With regard to the natural history of ovarian cysts discovered during pregnancy, it is believed that 10% will be operated on soon after diagnosis whilst a further 2% will require intervention later on in view of painful complications. A further 3% may be removed at caesarean section or in the puerperal period [1]. An average of half of the cysts removed have previously been noted to show neoplastic changes [1]. Surgery either open or laparoscopic has increasing risks with advancing pregnancy as it is a hypercoagulable state. Furthermore, although laparoscopic surgery has been performed in all trimesters of pregnancy, the risk of injury to the gravid uterus, poor visualization of the surgical fields, and preterm delivery are increased with advancing gestation [2]. It should be highlighted that ultrasound examination in the first and second trimester should not solely focus on fetal parameters but evaluate the cervix and adnexa. Ovarian cysts detected early can be managed promptly thus avoiding emergency procedures and reducing the risk of preterm delivery.

Benign dermoid cysts/teratomas are the most frequent ovarian tumors, with an incidence ranging from 5% to 25% of all ovarian neoplasms [3]. They are of germ cell origin and composed of multiple types of tissue. Torsion of the cystic contents and ovary may occur in them, thus leading to vascular infarction and necrosis. Torsion of the pedicle has been reported to be the most frequent complication, occurring in 16.1% of cases [3]. Traditional risk factors for ovarian torsion are increased ovarian size, ovarian tumors, ovarian hyperstimulation, and pregnancy [4–6].

Torsion of the ovary in the third trimester is rare as the compressive effect of the gravid uterus restricts the mobility of the ovarian pedicle. However this case clearly demonstrates that it can occur and needs to be considered as a differential diagnosis when patients present with an acute abdomen. Although conservative treatment has been proposed during pregnancy, surgical intervention is the treatment of choice once ovarian torsion is highly suspected [7].

Additionally this case highlights the difficulty in producing good quality radiological imaging of the pelvic organs in advanced pregnancy. Radiologists often have limited experience of pelvic imaging in the third trimester, so in all but the most experienced hand, a definitive diagnosis may not be forthcoming. This case serves to remind us of the importance of clinical acumen alongside diagnostic test as well as ensure that the correct incision is performed to ensure good surgical access. Furthermore, ultrasound scan examinations in early pregnancy should also address the cervix and the adnexa leading to early diagnosis and management of ovarian masses, thus avoiding later emergency situations and the possibility of preterm deliveries.
